# Using the Gene Ontology to Annotate Key Players in Parkinson’s Disease

**DOI:** 10.1007/s12021-015-9293-2

**Published:** 2016-01-29

**Authors:** R. E. Foulger, P. Denny, J. Hardy, M. J. Martin, T. Sawford, R. C. Lovering

**Affiliations:** Centre for Cardiovascular Genetics, Institute of Cardiovascular Science, University College London, London, UK; Department of Molecular Neuroscience, Institute of Neurology, University College London, London, UK; European Molecular Biology Laboratory, European Bioinformatics Institute (EMBL-EBI), Wellcome Trust Genome Campus, Hinxton, Cambridgeshire UK

**Keywords:** Annotation, database, Functional annotation, Gene ontology, High-throughput analysis, Parkinson’s disease

## Abstract

The Gene Ontology (GO) is widely recognised as the gold standard bioinformatics resource for summarizing functional knowledge of gene products in a consistent and computable, information-rich language. GO describes cellular and organismal processes across all species, yet until now there has been a considerable gene annotation deficit within the neurological and immunological domains, both of which are relevant to Parkinson’s disease. Here we introduce the Parkinson’s disease GO Annotation Project, funded by Parkinson’s UK and supported by the GO Consortium, which is addressing this deficit by providing GO annotation to Parkinson’s-relevant human gene products, principally through expert literature curation. We discuss the steps taken to prioritise proteins, publications and cellular processes for annotation, examples of how GO annotations capture Parkinson’s-relevant information, and the advantages that a topic-focused annotation approach offers to users. Building on the existing GO resource, this project collates a vast amount of Parkinson’s-relevant literature into a set of high-quality annotations to be utilized by the research community.

## Introduction

Parkinson’s disease is the second most common neurodegenerative disorder after Alzheimer’s disease. An increased understanding of Parkinson’s disease has resulted from the identification and characterization of genes that cause or influence the risk of developing this condition (Gasser et al. 2011). Much of this knowledge has accumulated from ‘big data’ analyses, such as genome wide association (GWA) studies, large-scale sequencing, proteomics and transcriptomic analyses. Researchers have the increasingly complex task of evaluating these large datasets to detect relevant pathways and regulatory networks, supported by resources including the Gene Ontology (GO) (Gene Ontology Consortium 2015), Reactome (Croft et al. 2014), KEGG (Kotera et al. 2012) and protein interaction databases (Kerrien et al. 2012).

GO is widely recognised as the gold standard bioinformatics resource for summarizing functional knowledge of gene products across all kingdoms of life. GO contains three ontologies describing the molecular functions of a gene product, the processes those actions are part of, and the cellular location(s) of the gene product. GO terms are then associated with a gene product to create an ‘annotation’. GO annotations are linked to an underlying reference, and include an evidence code indicating the assay, algorithm or decision supporting the annotation. A combination of manual and computational approaches are taken to create these annotations, and include summarising published research, inference by homology to related annotated gene products, and mapping of annotations from other biological resources such as Reactome, KEGG, and InterPro (Mitchell et al. 2015).

GO is frequently used in the analysis of high-throughput datasets, particularly for enrichment analyses where a gene-set of interest is analysed for over-represented GO terms, as demonstrated by Manzoni and colleagues in their examination of the LRRK2 interactome (Manzoni et al. 2015). Meaningful interpretation of such analyses depends on the quality of annotations that are available for the identified gene products, and for Parkinson’s datasets this approach has been hampered by insufficient functional annotation of many of the key affected neurological and immunological pathways, both of which are relevant to Parkinson’s disease (Holmans et al. 2013).

## Results and Discussion

The Parkinson’s GO Annotation Initiative began in January 2014 to address the neurological and immunological annotation deficit through the annotation of human proteins relevant to the risk and progression of Parkinson’s disease, principally by the expert curation of the biomedical literature. We aim to comprehensively curate the role of 800 human proteins within Parkinson’s-related processes by the end of 2016, and we are well on our way to our annotation target with over 1000 proteins already annotated, including 644 human proteins (as of August 2015). We consider a protein to be comprehensively annotated within a process when a curator is confident that the annotations cover all aspect of the protein’s function within that process. An overview of the full role of a protein within a process is typically gained from reading recent reviews. In practice, we have a limited time to annotate each protein, consequently we usually stop annotating a well-researched protein when annotation of additional papers leads to the repeated association of the same GO term to the protein. Proteins described in only a small number of papers will be annotated based on all available experimental evidence. Some proteins will be comprehensively annotated across multiple processes as we are taking a protein-based approach alongside our topic-based approach, as described below. One such fully-annotated protein is human PARK7 (DJ-1, Q99497), which (as of 6th August 2015) has 226 annotations in total, 135 of which were created by our project (Table 1 provides a selection of these annotations, note UniProtKB/Swiss-Prot protein IDs are used throughout this article). Furthermore, 22 PARK7 annotations were assigned semi-automatically by the Ensembl Compara pipeline (Cunningham et al. 2015) and eight of these derive from our manual annotations to the orthologous mouse Park7 entry (Q99LX0). Thus our project is increasing numbers of both manual and electronic annotations to improve coverage.

**Table 1:** Key annotations for human PARK7. A subset of annotations for human PARK7 (DJ-1, Q99497) adapted from QuickGO. Evidence code acronyms are IEA: Inferred from Electronic Annotation, IDA: Inferred from Direct Assay and IMP: Inferred from Mutant Phenotype. The ‘with’ field provides additional information such as the Ensembl identifier of the annotated protein used as a source of the orthology-based IEA annotation. A full set of annotations can be viewed in AmiGO or QuickGO

GO identifier	GO term name	Qualifier	Evidence	Reference	With	Assigned by
Process						
GO:0007005	mitochondrion organization		IEA	Ensembl Compara	Ensembl:ENSMUSP00000101299	Ensembl
GO:0033234	negative regulation of protein sumoylation		IDA	PMID:16731528		ParkinsonsUK-UCL
GO:0031397	negative regulation of protein ubiquitination		IDA	PMID:17015834		ParkinsonsUK-UCL
GO:0051444	negative regulation of ubiquitin-protein transferase activity		IDA	PMID:24899725		ParkinsonsUK-UCL
GO:0019243	methylglyoxal catabolic process to D-lactate via S-lactoyl-glutathione		IDA	PMID:22523093		ParkinsonsUK-UCL
GO:1903377	negative regulation of oxidative stress-induced neuron intrinsic apoptotic signaling pathway		IDA	PMID:15790595		ParkinsonsUK-UCL
GO:0051583	dopamine uptake involved in synaptic transmission		IEA	Ensembl Compara		Ensembl
GO:1903122	negative regulation of TRAIL-activated apoptotic signaling pathway		IMP	PMID:21785459		ParkinsonsUK-UCL
GO:1903197	positive regulation of L-dopa biosynthetic process		IMP	PMID:16731528		ParkinsonsUK-UCL
GO:0050821	protein stabilization		IMP	PMID:17015834		ParkinsonsUK-UCL
GO:0010273	detoxification of copper ion		IMP	PMID:23792957		UniProt
GO:0050787	detoxification of mercury ion		IMP	PMID:23792957		UniProt
GO:0045944	positive regulation of transcription from RNA polymerase II promoter		IMP	PMID:19703902		ParkinsonsUK-UCL
Function						
GO:0036470	tyrosine 3-monooxygenase activator activity		IDA	PMID:19703902		ParkinsonsUK-UCL
GO:0036478	L-dopa decarboxylase activator activity		IDA	PMID:19703902		ParkinsonsUK-UCL
GO:1990422	glyoxalase (glycolic acid-forming) activity		IDA	PMID:22523093		ParkinsonsUK-UCL
GO:0008233	peptidase activity		IDA	PMID:20304780		UniProt
GO:0016532	superoxide dismutase copper chaperone activity		IDA	PMID:24567322		ParkinsonsUK-UCL
GO:0042803	protein homodimerization activity		IDA	PMID:24144264		ParkinsonsUK-UCL
GO:1903135	cupric ion binding		IDA	PMID:24567322		ParkinsonsUK-UCL
GO:1903136	cuprous ion binding		IDA	PMID:24567322		ParkinsonsUK-UCL
GO:0045340	mercury ion binding		IDA	PMID:23792957		UniProt
GO:0003729	mRNA binding		IDA	PMID:18626009		ParkinsonsUK-UCL
GO:0003690	double-stranded DNA binding	NOT	IDA	PMID:22683601		ParkinsonsUK-UCL
GO:0003697	single-stranded DNA binding	NOT	IDA	PMID:22683601		ParkinsonsUK-UCL
Component						
GO:0005829	cytosol		IDA	PMID:14662519		ParkinsonsUK-UCL
GO:0005634	nucleus		IDA	PMID:22683601		ParkinsonsUK-UCL
GO:0016605	PML body		IDA	PMID:22683601		ParkinsonsUK-UCL
GO:0070062	extracellular exosome		IDA	PMID:19056867		UniProt
GO:0005739	mitochondrion		IDA	PMID:15944198		ParkinsonsUK-UCL
GO:0005758	mitochondrial intermembrane space		IEA	Ensembl Compara	Ensembl:ENSMUSP00000101299	Ensembl
GO:0005759	mitochondrial matrix		IEA	Ensembl Compara	Ensembl:ENSMUSP00000101299	Ensembl

Given the vast amount of literature available on Parkinson’s disease, our first task was to define an annotation priority set, and below we discuss the steps taken to prioritise proteins, publications and cellular processes. Our high priority protein set (https://www.ucl.ac.uk/functional-gene-annotation/neurological/projects/high-priority-genes) currently includes 48 proteins encoded either by familial Parkinson’s disease genes or by candidate genes identified in GWA studies. An extended priority list (http://www.ebi.ac.uk/QuickGO/GProteinSet?id=ParkinsonsUK-UCL) also contains proteins that interact with the high priority set, as identified by IntAct (Porras et al. 2015), and proteins that participate in Parkinson’s-relevant processes, as described below.

A list of pathways and processes relevant to Parkinson’s disease was assembled based on publications and discussions with researchers. We took a process-based approach to annotation alongside our protein-based strategy, to ensure that all proteins within a Parkinson’s-relevant process are annotated, and not just proteins with an already established link to the disease. The process priority list includes mitochondrial processes (such as mitophagy and mitochondrial fusion and fission), response to cellular stresses (including oxidative stress, endoplasmic reticulum (ER) stress and nitrosative stress), Wnt signaling, lysosomal pathways, dopamine metabolism, dopamine transport, autophagy and synaptic transmission. We are annotating all key players in these processes to capture multiple aspects of Parkinson’s disease and allow examination of the cellular networks that underlie the condition. We define a key player as a protein that has been shown to be critical to a process or regulation of a process, information we acquire primarily through literature curation but also from attending meetings in the field and communicating with researchers. In addition, we are prioritising curation of Parkinson’s UK-funded research articles. Furthermore, our priority lists remain dynamic and are expanded to reflect new knowledge and scientific advancements.

Thus far, we have manually curated nearly 300 publications, and generated over 4000 manual annotations, including over 2700 annotations to human proteins (as of 6th August 2015). Our annotation focuses on human proteins, but given the enormous knowledge contribution from model organism data (Magen and Chesselet 2010; Pienaar et al. 2010), we also annotate other organisms with a priority on mouse, rat and fly. GO data is then transferred to orthologous human proteins through the Ensembl-Compara pipeline or, in the absence of automated methods, we manually transfer GO annotations between orthologs using the ISS (Inferred from Sequence or structural Similarity) evidence code.

The role of a gene product can vary depending on its environment or interactors, and in a GO Consortium-led approach (Huntley et al. 2014), we extend the core GO annotation to accommodate additional context, such as the cell type the process occurs in, or the substrate of a catalytic activity. For example, we capture that PML (P29590) and PARK7 (DJ-1, Q99497) interact under conditions of oxidative stress (Kim et al. 2012) by extending the ‘protein binding’ (GO:0005515) annotation with the statement “*happens*_*during* cellular response to oxidative stress (GO:0034599)”. By referencing the cell-type and anatomy ontologies (Bard et al. 2005; Mungall et al. 2012) we annotate that HERPUD1 (HERP, Q15011) is located in the ER of a neuron in the substantia nigra (Slodzinski et al. 2009) by including “*part*_*of* CL:0000540, *part*_*of* UBERON:0002038” in the annotation. One of the biggest benefits of providing this contextual information is the ability to record the target of a protein’s activity, e.g. capturing that murine Nfe2l2 (Nrf2, Q60795) is a phosphorylation substrate of Eif2ak3 (Perk, Q9Z2B5) during the Unfolded Protein Response (UPR) (Cullinan et al. 2003). In this way we can link annotations together to provide the full picture of a pathway or process, and in the future GO browsers will support more sophisticated queries and analyses allowing the extraction of, for example, all EIF2AK3 (PERK) substrates.

To ensure annotations are of a high quality, we annotate to GO Consortium guidelines that have been rigorously developed by trained curators. Internal and Consortium quality-checking procedures, including peer-review, ensure that annotations conform to these rules and are appropriate to the taxon of the organism being annotated (Deegan née Clark et al. 2010). A long-term approach to maintaining annotation quality comes from the Phylogenetic Annotation Inference Tool (PAINT) project (Gaudet et al. 2011) whereby annotations are inferred through the phylogenetic tree. Curator input into the annotation propagation ensures consistent annotation across orthologs and highlights any potential anomalies or errors. Our users and authors offer another layer of quality control; we correspond with authors during curation to clarify ambiguities (for example the species origin of construct sequences). Furthermore, when paper annotation is complete, we regularly notify the authors who can check that our annotations accurately represent the published data.

### Ontology Improvements

**Fig. 1:**
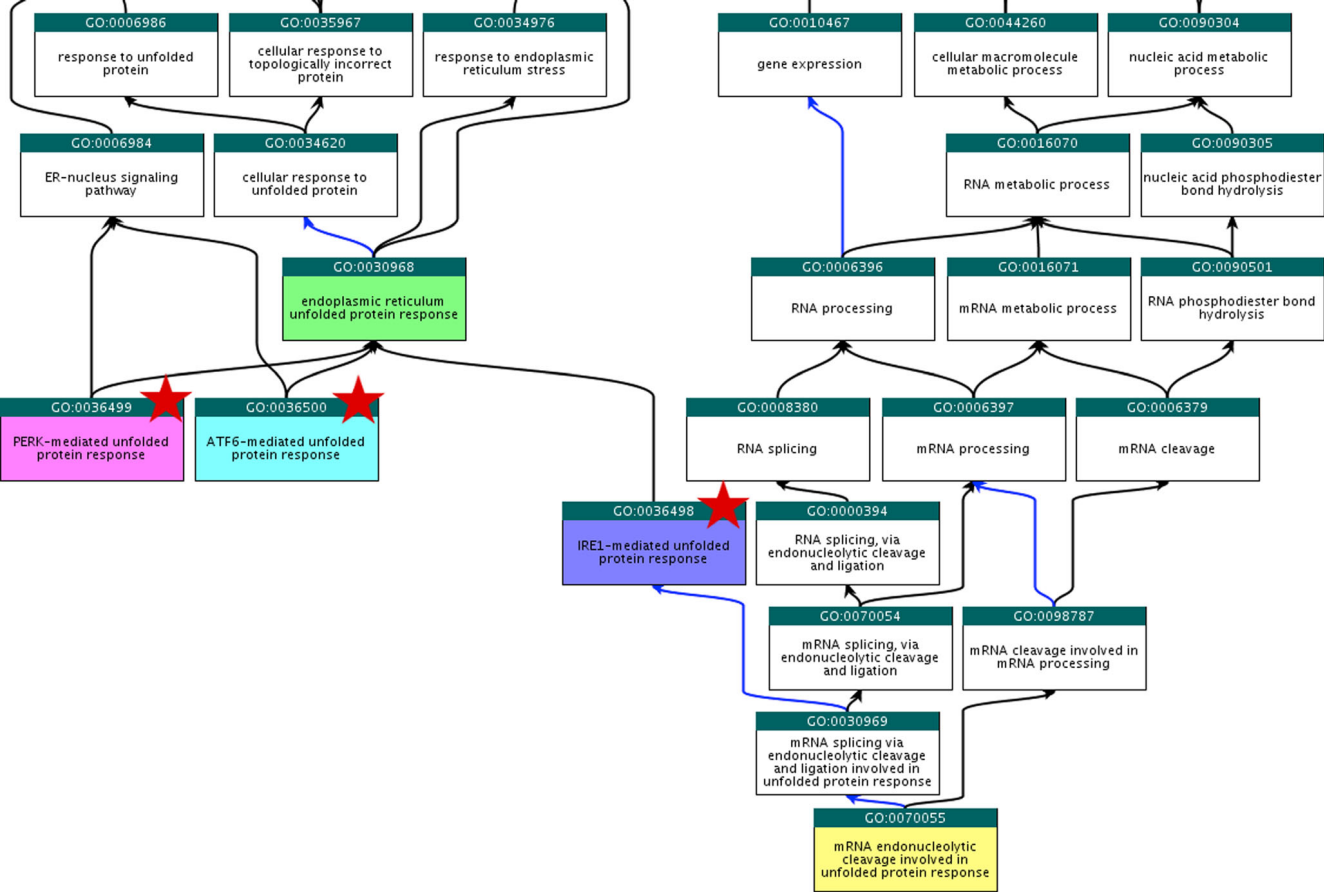
Representation of the Unfolded Protein Response (UPR) in the Gene Ontology. To better represent the endoplasmic reticulum UPR (GO:0030968: in *green*) within the Gene Ontology, we created new terms (denoted by *red stars*) for ‘IRE1-mediated unfolded protein response’ (GO:0036498), ‘PERK-mediated unfolded protein response’ (GO:0036499) and ‘ATF6-mediated unfolded protein response’ (GO:0036500), covering the three main branches of the mammalian UPR. GO:0070055 (in *yellow*) describes IRE1-mediated non-spliceosomal cleavage of the XBP1 mRNA in mammals and Ire1p-mediated cleavage of the HAC1 mRNA in yeast. Additional terms exist in this node but are not shown for conciseness. *Black arrows* denote *is*_*a* relationships between terms and *blue arrows* denote *part*_*of* relationships between terms. Image adapted from QuickGO

Ontology development goes hand-in-hand with annotation, thus GO is being continually updated and expanded. A considerable benefit of a topic-based curation approach is that expert curators can apply their accumulated knowledge of a biological research area to improve the ontology terms and structure. To date, our project has contributed over 240 new GO terms across all three ontology nodes including ‘CHOP-C/EBP complex’ (GO:0036488), ‘ERAD pathway’ (GO:0036503), ‘chaperone-mediated autophagy’ (GO:0061684), ‘L-dopa biosynthetic process’ (GO:1903185) and ‘tyrosine 3-monooxygenase activator activity’ (GO:0036470), thereby enabling more descriptive annotations to be created. Using the UPR as an example; the endoplasmic reticulum UPR is one pathway by which cells deal with unfolded and misfolded proteins in the ER to restore the cell to its resting state. ER stress has extensive links to Parkinson’s disease (Varma and Sen 2015) and is therefore a project priority topic. To expand the ontology in this node, we created new terms to represent the three main branches of the mammalian UPR (Fig. 1). Previously, the transcription factor ATF4 (P18848) had been annotated to ‘endoplasmic reticulum unfolded protein response’ (GO:0030968). While this annotation remains true, it does not capture how ATF4 fits into this process. With our creation of new UPR terms, we were able to associate the more descriptive term ‘PERK-mediated unfolded protein response’ (GO:0036499) with ATF4. GO terms mentioning gene products are the exception but where they exist, the name reflects the gene or protein name most commonly used in the research field (e.g. PERK) and is designed to be species-neutral wherever possible. Synonyms including species-specific nomenclature (e.g. EIF2AK3) are added when required to capture additional information and improve search capabilities. Ontology construction requires considerable curator input, consequently we have created over 180 of our new GO terms using the TermGenie tool (Dietze et al. 2014), which enables formally specified design patterns and templates to appropriately place and define each new term.

### Call for Community Contributions

To make the greatest impact in the area of Parkinson’s disease, it is important that we consult experts in the field to ensure our GO annotations and terms are accurately capturing current knowledge. Thus we have established a scientific advisory panel for consultation and we interact with researchers and authors during curation, as described above. When curating a topic, we also refer to Reactome (Croft et al. 2014) and the Parkinson’s disease map (Fujita et al. 2014) to align our efforts with these manually-curated resources of cellular pathways and Parkinson’s-related mechanisms, respectively. We have held discussions with the scientists responsible for these resources to standardise annotations and Parkinson’s-related GO terms, and we plan to create further reciprocal links as our project progresses. We welcome feedback from the community, and there are many additional ways in which researchers can be involved with the project, or be kept informed about developments. Please contact us (goannotation@ucl.ac.uk) to:

i) Subscribe to our quarterly newsletter.
ii) Extend our gene priority lists by supplying us with details of key proteins.
iii) Suggest gene annotations that are currently missing, or literature that requires curation.

Furthermore, our project progress can be followed on our Twitter account (@UCLgene) and the go-friends email list (http://geneontology.org/page/go-mailing-lists) can be subscribed to stay informed about all GO projects.

## Conclusions

With this project we are providing an integrated, comprehensive set of annotations to describe proteins implicated, or predicted to play a role, in Parkinson’s disease. Our project will specifically build on the efforts of the Toronto Alzheimer’s disease annotation project (Patel et al. 2015), which is focused on using GO to identify proteins involved in Alzheimer’s disease through imaging genetics studies. To enhance the functionality of GO in their analyses, they created over 30 new relevant GO terms, and nearly 200 GO annotations to 15 human proteins (http://www.ebi.ac.uk/QuickGO/GAnnotation?tax=9606&source=Alzheimers_University_of_Toronto) with some notable overlap in prioritized proteins (e.g. Tau). There are a growing number of focused GO annotation projects, such as those covering the cardiovascular and transcription domains (Alam-Faruque et al. 2011; Tripathi et al. 2013), and these combined efforts are, together with the major GO Consortium members groups, improving the breadth and depth of the GO annotations available.

Previous GO Consortium projects have demonstrated the benefit of a topic-based annotation approach with notable improvements of the interpretation of relevant datasets (Alam-Faruque et al. 2014), and we envisage that our project will have a similar impact on the analysis of many neurobiological datasets, not only those related to Parkinson’s disease. Many neurodegenerative diseases share underlying cellular and molecular mechanisms, and the majority of the processes we have prioritised for annotation are relevant to other diseases. Disruption of the autophagy machinery, for example, can lead to a number of neurological disorders (Nah et al. 2015) including Parkinson’s disease, Alzheimer’s disease, Huntington’s disease, Amyotrophic lateral sclerosis (ALS) and Multiple sclerosis, and beyond neurodegeneration autophagy has been linked to cancer, aging, liver and muscle disorders, and infection by pathogens (Shintani and Klionsky 2004). Dys-regulated Wnt signalling has also been suggested to underlie many neurological conditions including Alzheimer’s disease (Sadigh-Eteghad et al. 2015), autism and schizophrenia (reviewed in Berwick and Harvey 2014), and abnormal CDK5 signaling has a role in the pathogenesis of various neurodegenerative disorders including Parkinson’s and Alzheimer’s diseases, disrupting proteins including α-synuclein (ASN) and β-amyloid peptide (Aβ) (Wilkaniec et al. 2015). Lysosomal transport was prioritised for curation based on its relevance to Parkinson’s disease but it has wider implications, with lysosomal storage disorders and neurodegenerative disorders sharing a dysfunctional cellular transport network (Boland and Platt 2015).

Stress-insult is a common theme across human health, with the antioxidant PARK7 (DJ-1) playing a central role in protecting against oxidative stress in cancer, cardiovascular disease and neurodegeneration (Chan and Chan 2015). ER-stress and particularly ERAD is linked to more than 60 diseases (Guerriero and Brodsky 2012; Yoshida 2007), including Cystic fibrosis (Kerbiriou et al. 2007) and bipolar disorder (Pfaffenseller et al. 2014). Our prioritized proteins are also implicated in additional disorders; a search in OMIM (http://omim.org/) reveals that mutations in *MAPT* encoding the microtubule-associated protein Tau are linked to a number of neurological disorders including Frontotemporal dementia (FTD), Progressive supranuclear palsy 1 (PSNP1), and of course Alzheimer’s disease.

These examples are by no means exhaustive but aim to demonstrate how our improved annotations and additions to the GO structure will feed into the interpretation of datasets across a wide range of diseases, not only within the neurodegenerative spectrum, but also extending to other areas of health and disease. More manual annotations will mean that users will get more meaningful functional data analysis, with potentially more specific terms being significantly enriched in transcriptomic or proteomic studies. Furthermore, GO is already being used to improve GWAS identification of risk SNPs (Holmans et al. 2013), and improving GO for Parkinson’s disease is likely to further aid this approach, for a range of neurodegenerative diseases.

Combining GO with external resources further enhances its applications: Cytoscape (Shannon et al. 2003) enables our improved GO annotations to be combined with interaction data captured by IntAct, so researchers can visualize and manipulate protein interaction networks overlaid with significantly enriched GO processes. These networks may be used to predict ‘hubs’, key positions at which the whole network may be perturbed, and thus inform future research. Tools are currently being developed which will allow users to create complex queries based on the cross-links between GO and the chemical ontology ChEBI (Hill et al. 2013; Mungall et al. 2011), and the Parkinson’s-relevant annotations. Thus it will be possible to identify all gene products involved in dopamine processes in unrelated parts of the GO graph, such as dopamine transport, dopamine metabolism and dopamine binding, or all proteins involved in the metabolism of dopaminergic agents. We hope that our resource will benefit neurobiology researchers and scientists, and we encourage you to contact us with your questions or suggestions.

## Information Sharing Statement

All of the annotations associated with our prioritised Parkinson’s-relevant proteins are freely available for viewing or download in QuickGO (http://www.ebi.ac.uk/QuickGO/, RRID:SCR_004608) (Binns et al. 2009) using the ‘Gene Product ID’ filter ‘ParkinsonsUK-UCL’ or in the GO Consortium browser, AmiGO (http://amigo.geneontology.org/, RRID:SCR_002143) (Carbon et al. 2009). In addition, these annotations and ontologies are uploaded to major biological knowledgebases such as Ensembl, NCBIGene and UniProtKB, and are available for download and local installation via ftp sites. Users can perform enrichment analysis on neurological datasets using a number of GO-supported enrichment tools (http://geneontology.org/page/go-enrichment-analysis).
